# Biodegradation and biodetoxification of *Fusarium* mycotoxins by *Sphaerodes mycoparasitica*

**DOI:** 10.1186/s13568-017-0446-6

**Published:** 2017-07-06

**Authors:** Seon Hwa Kim, Vladimir Vujanovic

**Affiliations:** Department of Food and Bioproduct Sciences, University of Saskatchewan, 51 Campus Drive, Saskatoon, SK S7N 5A8 Canada

**Keywords:** Mycotoxins, *Fusarium*, Biocontrol, Biodegradation, Biotransformation, Biodetoxification

## Abstract

A fungus *Sphaerodes mycoparasitica* SMCD 2220-01 is a host specific mycoparasite against plant pathogenic *Fusarium* species. *Fusarium* spp. are producing a plethora of mycotoxins including zearalenone (ZEN), deoxynivalenol (DON) and its acetylated derivatives, 3-acetyl-deoxynivalenol (3-ADON) and 15-acetyl-deoxynivalenol (15-ADON). The SMCD 2220-01 strain substantially reduced DON, 3-ADON, 15-ADON, and ZEN production capacity in co-culture system. Degradation and detoxification of the pure mycotoxins were also achieved when exposed to SMCD 2220-01 in shake flasks. The thin layer chromatography (TLC) combined with high performance liquid chromatography–electrospray ionization-high resolution mass spectrometry (HPLC–ESI–HRMS) revealed that the amount of mycotoxins exposed to SMCD 2220-01 was considerably reduced compared to control. ZEN level was decreased by 97%, while zearalenone sulfate ([M−H+SO_3_]^−^ at *m/z* 397.1052 C_18_H_21_O_8_S_1_) was detected as a metabolite of ZEN converted to less toxic molecule by the mycoparasite. Further, the mycoparasite appeared to degrade DON, 3-ADON, and 15-ADON by 89, 58, and 72%, respectively. The deoxynivalenol sulfate ([M−COCH_3_+SO_3_−CH_2_O]^−^ at *m/z* 345.2300 C_14_H_17_O_8_S_1_) was detected as a less toxic metabolic product of DON and 3-ADON. These findings report the SMCD 2220-01 effectiveness to lower mycotoxins-producing capacities of *Fusarium*, degrade pure mycotoxins and transform them to less toxic metabolites, opening new opportunities for research and innovation for detoxification of mycotoxins.

## Introduction

*Fusarium graminearum, Fusarium culmorum* and *Fusarium avenaceum* are the causal agent of *Fusarium* head blight (FHB) of small grain cereals in fields worldwide (Kim and Vujanovic 2016). These *Fusarium* species can produce various secondary metabolites including toxins such as zearalenone (ZEN), deoxynivalenol (DON/vomitoxin), 3-acetyl-deoxynivalenol (3-ADON), 15-acetyl-deoxynivalenol (15-ADON) and aurofusarin (AUR). ZEN, a member of the resorcyclic acid lactone family, is a known hydrophobic mycotoxin produced by *F. graminearum* and *F. culmorum* (Caldwell et al. 1970; Katzenellenbogen et al. 1979). The toxicity of ZEN is mainly derived from its lactone ring and free C-4 hydroxyl group (El-Sharkawy and Abul-Hajj 1988a). ZEN contamination found in maize and wheat grains, food and feed, poses a threat to health of humans and animals due to its similar chemical structure to estrogen (Iqbal et al. 2014; Shier et al. 2001). DON, 3-ADON, and 15-ADON, belonging to Type B trichothecenes, in which the 12,13-epoxy ring is essential for their cytotoxicity (Zhou et al. 2008); it was revealed as inhibitor of eukaryotic protein synthesis as well as RNA and DNA synthesis (Ehrlich and Daigle 1987; Hussein and Brasel 2001; Middlebrook and Leatherman 1989). The toxicity of DON and its derivatives, 15-ADON and 3-ADON, were demonstrated by using the 5-bromo-2′-deoxyuridine incorporation assay assessing DNA-synthesis (Sundstøl Eriksen et al. 2004), in which there may be a link between degradation process and resistance to the mycotoxins.

Mycotoxin-degrading microbes have been isolated mainly from agriculture crops and plant production environments. *Sphaerodes mycoparasitica* SMCD 2220-01 is the fungus originally isolated from wheat and asparagus fields in association with *F. graminearum, F. oxysporum* and *F. avenaceum* (Vujanovic and Goh 2009). SMCD 2220-01 shown mycoparasitic lifestyle and is promising biological control agent against mycotoxin producing *Fusarium* pathogens (Vujanovic and Goh 2010; Kim and Vujanovic 2016). In addition to the biocontrol effect, SMCD 2220-01 was effective in reducing AUR mycotoxin production in red pigmented *Fusaria* by down-regulating AUR gene expression (Vujanovic and Goh 2011, Vujanovic et al. 2017). Although the mycoparasite showed efficacy in moderating DON, 3-ADON, 15-ADON, and production in Fusaria (Vujanovic and Chau 2012), the background mechanism of mycoparatism that occur at the molecular level is still unknown. Therefore, we hypothesized that SMCD 2220-01 effectiveness to reduce mycotoxin-producing capacities of *Fusarium* in co-culture is also related to its mycoparasitic ability to degrade or detoxify the substrates or media contaminated with *Fusarium* mycotoxins. The shifts in ZEN, DON, 3-ADON and 5-ADON was evaluated using *Fusarium*-standard thin layer chromatography (TLC) (Vujanovic et al. 2012) combined with high performance liquid chromatography–electrospray ionization–high resolution mass spectrometry (HPLC–ESI–HRMS) which is characterized with superior performance and sensitivity to discover masked or modified mycotoxins (De Boevre et al. 2016).

## Materials and methods

### Fungal cultures, chemicals and media

**Fig. 1:**
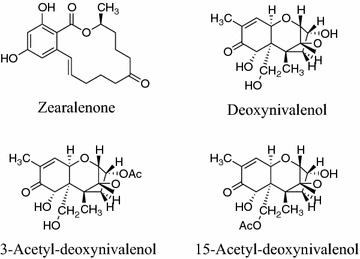
Chemical structure of the mycotoxin zearalenone (ZEN), deoxynivalenol (DON), 3-acetyl-deoxynivalenol (3-ADON), and 15-acetyl-deoxynivalenol (15-ADON)

In this study, the mycoparasitic biocontrol *Sphaerodes mycoparasitia* SMCD 2220-01 strain deposited in IDAC under accession number 301008-01 (Public Health Agency of Canada—International Depositary Authority of Canada Collection, Winnipeg, Canada) has been used for decomposition and detoxification of *Fusarium* mycotoxins. Zearalenone (ZEN), deoxynivalenol (DON), 3-acetyl-deoxynivalenol (3-ADON), and 15-acetyl-deoxynivalenol (15-ADON) shown in Fig. 1 were purchased from Sigma-Aldrich Canada Ltd., Oakville, ON. HPLC grade organic solvents were purchased from Fisher Scientific. The stock solutions of each of mycotoxins were prepared by dissolving each mycotoxin in acetonitrile. Potato dextrose broth (PDB, BD Difco) and agar (PDA) were used for maintaining SMCD 2220-01 and biodegradation experiments.

### Evaluation of the efficacy of the mycotoxin-degrading capacity

In order to induce degradation capacity of *S. mycoparasitica,* SMCD 2220-01 strain was grown on the cellulose membrane placed on PDA amended with 1 mg L^−1^ of each of mycotoxins. The mycelium of the induced SMCD 2220-01 was inoculated in 5 mL of PDB and incubated at 23 °C on a rotary shaker at 120 rpm for 3 days in the dark condition. The pre-cultured SMCD 2220-01 was incubated with 2 mg L^−1^ of ZEN, DON, 3-ADON, and 15-ADON, respectively. A non-treated with each mycotoxin and inoculated with SMCD 2220-01 in medium (only SMCD 2220-01) was used to exclude metabolites of SMCD 2220-01. A treated with each mycotoxin but not inoculated with SMCD 2220-01 in medium (only each mycotoxin) was prepared to check natural decomposition of mycotoxins. A medium, PDB as a control was used to exclude impurities from the medium itself. All the cultures were incubated on a rotary shaker at 120 rpm at 23 °C in the dark condition. Cultures were harvested at 1, 2, and 3 weeks after the addition of mycotoxins. The harvested cultures were filtered by Whatman filter paper Grade 2 to remove mycelia. The culture filtrates were used for extraction of the residual mycotoxins by liquid–liquid partition.

### Detection and semi-quantification of mycotoxins by TLC

The culture filtrates were extracted by 5 mL of ethyl acetate (EtOAc) and evaporated to dryness. The EtOAc extracts were dissolved in 200 µL of chloroform for thin layer chromatography (TLC) (Bejaoui et al. 2006; Garda-Buffon and Badiale-Furlong 2010; Teniola et al. 2005). For four mycotoxins, 4, 8, and 8 µL of the final extracts of SMCD 2220-01, SMCD 2220-01 treated with mycotoxins, and mycotoxins in medium in sequence were spotted, along with authentic mycotoxin standards (1 µL of 1000 ppm) on the base of an aluminum TLC silica gel 60 F_254_ plate. The separation process was performed by using a mixture of dichloromethane and methanol with optimized developing times (95:5 for ZEN, 15-ADON, and 3-ADON and 93:7 for DON) as a mobile phase for developing the TLC plate (Abbas et al. 1984). Further, the developed TLC plate was dried and then visualized by charring solutions after checking under ultra violet light if needed. To interpret TLC spots, the relative mobility or retention factor (R_f_) was calculated by the following equation (Eq. 1).

1 $${\text{R}}_{\text{f}} {\text{ = Distance from start to center of substance spot}}/{\text{distance from start to solvent front}}$$

Semi-quantification of TLC spots for residual mycotoxins was achieved through densitometry analysis using Image J software. Image J software is available online at http://rsbweb.nih.gov/ij/plugins/index.html and provides an easy access to extract the area occupied by a specific color. Data are the mean of three replicate with error bars representing standard deviation analyzed by one-way analysis of variance (ANOVA) Tukey’s HSD (*P* < 0.05). Percent degradation of the mycotoxins by SMCD 2220-01 was calculated by using the equation (Eq. 2).

2 $${\text{Degradation rate (\%)}} = \left( { 1- A/A_{\text{C}} } \right) \times 100\;({\text{\%)}}$$

where *A* is the area of residual mycotoxin in the samples and *A*_c_ is the area of mycotoxin in the control (mycotoxin in medium).

### Confirmation of mycotoxin quantification by HPLC–ESI–HRMS

In order to confirm the quantification of residual mycotoxins and to elucidate transformants of mycotoxins by SMCD 2220-01, HPLC**–**ESI**–**HRMS was performed on an Agilent 1100 series high-performance liquid chromatography (HPLC) system equipped with an automatic injector, quaternary pump, degasser, and a diode array detector (DAD, wavelength range 190–600 nm) connected to a Qstar XL systems Mass Spectrometer (Hybrid Quadruple-TOF LC/MS) with turbospray electrospray ionization (ESI) source. Chromatographic separations were carried out using Eclipse XDB-C-18 column (5 µm particle size silica, 150 × 4.6 mm I.D.). All the extracted samples were dissolved in acetonitrile. Authentic mycotoxins (2, 4, 8, and 10 µL of 100 ppm) were used for generation of standard curves, as well as confirmation of ionization patterns of each mycotoxin for detection.

The mobile phase consisted of a linear gradient of 0.1% formic acid in water and 0.1% formic acid in methanol (95:5 in 5 min, to 80:20 in 25 min, to 50:50 in 35 min, to 25:75 in 40 min, to 5:95 in 45 min) and a flow rate of 0.1 mL min^−1^. Data acquisition was carried out either positive or negative polarity mode for LC run (DON, 3-ADON, and ZEN on negative mode and 15-ADON on positive mode). Data processing was conducted by Analyst QS Software. Percent degradation of the mycotoxins by SMCD 2220-01 was calculated by using the equation (Eq. 3).

3 $${\text{Degradation rate (\%)}} = \left( { 1- C/C_{\text{C}} } \right) \times 100\;(\%)$$

where *C* is the residual concentration of mycotoxin in the sample (mg L^−1^) and *C*_c_ is the concentration of mycotoxin (mg L^−1^) in the control (mycotoxin in medium).

### Statistical analysis

Data are the mean of three replicate with standard deviation. One-way analysis of variance (ANOVA) Tukey’s HSD was used to test whether each of residual mycotoxins in samples and controls based on TLC was significantly different (*P* < 0.05).

## Results

### Thin layer chromatography (TLC)

**Fig. 2:**
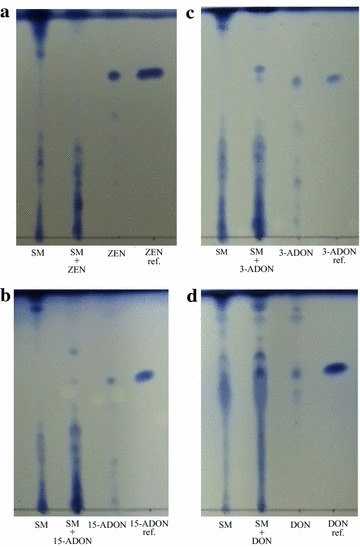
Thin layer chromatograms of extracts of culture filtrates at 3 week incubation supplemented with 2 mg L^−1^ of ZEN (**a**), 15-ADON (**b**), 3-ADON (**c**), and DON (**d**). From the *left side*, each of *lanes* indicates the SMCD 2220-01 only, SMCD 2220-01 treated with each mycotoxin, each mycotoxin in medium, and authentic mycotoxin as a reference

TLC analysis indicated the different level of residual mycotoxins and metabolites of extracts of culture filtrate derived from SMCD 2220-01 at 1, 2, and 3 weeks old cultures after the addition of each mycotoxin (data at 3 weeks shown in Fig. 2). R_f_ value of ZEN, 15-ADON, and 3-ADON was 0.69, 0.58, and 0.68 in the solvent system (95% dichloromethane and 5% methanol with developing 2, 4, and 4 times), respectively. 15-ADON and 3-ADON showed transformants as separate spots. R_f_ value of DON was 0.63 in the solvent system (93% dichloromethane and 7% methanol with developing 5 times). It seemed that DON with the transformants by SMCD 2220-01 was masked due to the similar polarity between DON and the transformants.

**Fig. 3:**
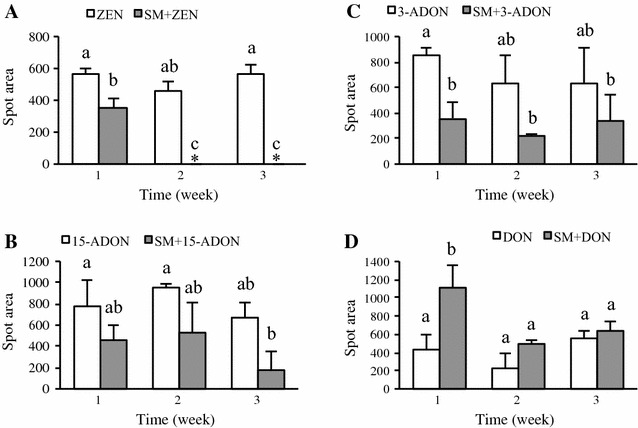
Densitometry analysis for the residual mycotoxin levels at 1, 2, and 3 weeks after addition of each mycotoxin based on spot area of TLC. Data are the mean of three replicate with *error bars* representing standard deviation analyzed by ANOVA Tukey’s HSD (*P* < 0.05). The *same letters above the error bars* do not differ significantly at *P* < 0.05. ZEN (**A**), 15-ADON (**B**), 3-ADON (**C**), and DON (**D**). Each mycotoxin in medium *open square*; SMCD 2220-01 with each mycotoxin in medium *closed square*; *no residual ZEN

Based on spot areas at R_f_ value for each mycotoxin, densitometry analysis allowed us to check efficacy of SMCD 2220-01 to decrease in ZEN by 38, 100, 100%, 15-ADON by 37, 45, 74%, 3-ADON by 37, 58, 63% at 1, 2, and 3 weeks after the addition of each mycotoxin, respectively (Fig. 3A–C). However, DON could not be analyzed by TLC due to the similar polarity between DON and the transformants or the metabolites (Fig. 3D). In overall, through TLC, SMCD 2220-01 showed the most effective degradability on ZEN among other mycotoxins. In addition to ZEN, HPLC**–**ESI**–**HRMS analysis revealed also DON degradation products.

### HPLC–ESI–HRMS analysis

**Fig. 4:**
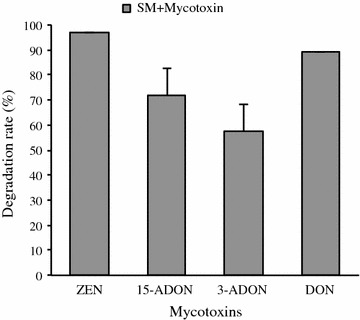
Degradation rate (%) of ZEN, 15-ADON, 3-ADON, and DON by SMCD 2220-01 at 3 weeks incubation. Values are the mean of three replicate and standard deviation

Mycotoxin detection and quantification as well as qualification of residual mycotoxins in extracts of culture filtrate for each mycotoxin treatment exposed to SMCD 2220-01 were confirmed by extracted ion chromatograms (XIC) through HPLC**–**ESI**–**HRMS analysis. It was shown that SMCD 2220-01 degrades 97, 72, 58, and 89% of ZEN, 15-ADON, 3-ADON, and DON at 3 weeks incubation in PDB after the addition of the mycotoxins, respectively (Fig. 4). The trend of mycotoxins degradation ability of SMCD 2220-01 indicated by LC–MS was fairly similar to that by TLC analysis, except for DON. In case of DON, XIC allowed us to calculate residual DON successfully and overcome TLC limitations.

**Fig. 5:**
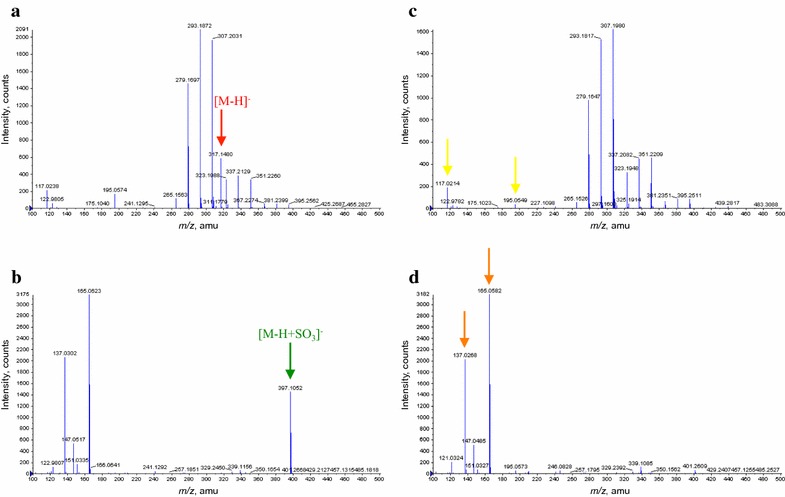
Representative mass spectra of extracts of culture filtrates analyzed by ESI–TOF–MS in negative-ion mode for ZEN treatments and controls. ZEN in PDB (**a**), SMCD 2220-01 treated with ZEN in PDB (**b**), PDB (**c**), and SMCD 2220-01 in PDB (**d**)

The extract of ZEN in PDB showed a peak of [M−H]^−^ at *m*/*z* 317.1480 identified as ZEN, while the extract of culture filtrate of SMCD 2220-01 treated with ZEN in PDB showed a peak at *m*/*z* 397.1052 of in negative-ion mode (Fig. 5a, b). The ZEN degradation product, [M−H+SO_3_]^−^ at *m*/*z* 397.1052 compound was identified as detoxified compound (C_18_H_21_O_8_S)^−^ according to Plasencia and Mirocha (1991). The difference of mass unit (only 9.0 millimass) between the observed mass (397.1052) and calculated mass (397.0962) may result from the electron-withdrawing effect of the sulfate group and by affected by dissolving solvent (Barron et al. 1988). It is likely that two peaks at *m*/*z* 195.0549 and 117.0214 are related with PDB compositions (Fig. 5c). The peaks at *m*/*z* 137.0268 and 165.0582 seem to relate with metabolite(s) of SMCD 2220-01 (Fig. 5d).

**Fig. 6:**
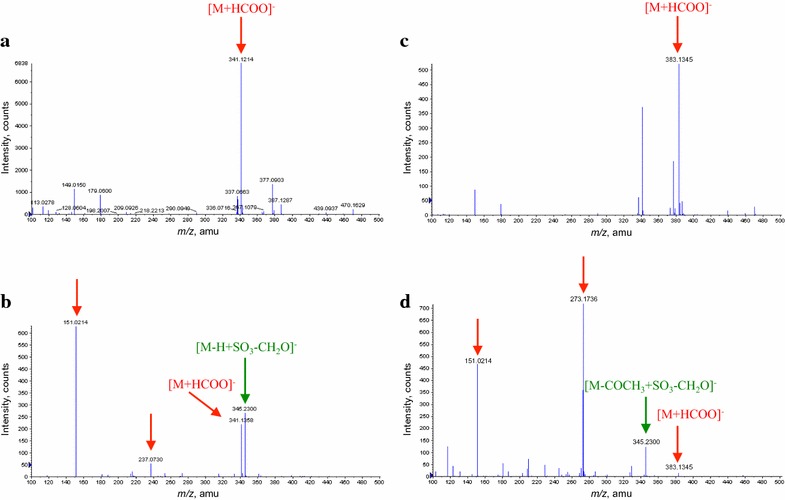
Representative mass spectra of extracts of culture filtrates analyzed by ESI–TOF–MS in negative-ion mode for DON and 3-ADON treatments. DON in PDB (**a**), SMCD 2220-01 treated with DON in PDB (**b**), 3-ADON in PDB (**c**), and SMCD 2220-01 treated with 3-ADON in PDB (**d**)

The extracts of culture filtrates from both DON in PDB and SMCD 2220-01 treated with DON in PDB showed peaks of [M+HCOO]^−^ at *m*/*z* 341.1214 and 341.1358 in negative-ion mode (Fig. 6a, b), identified as DON. It may be that peaks at *m*/*z* 151.0214, 237.0730, and 345.2300 are related with DON degradation product by SMCD 2220-01 or its fragment or metabolite of SMCD 2220-01 induced by DON (Fig. 6b). Especially, a peak at *m*/*z* 345.2300 might be considered, as a fragment ion which lost CH_2_O from CH_2_OH group attached to the carbon at the C-6 position of deoxynivalenol-3-sulfate (Warth et al. 2014).

The extracts of culture filtrates from both 3-ADON in PDB and SMCD 2220-01 treated with 3-ADON in PDB showed a peak of [M+HCOO]^−^ at *m*/*z* 383.1345 in negative-ion mode (Fig. 6c, d), identified as 3-ADON. It is likely that peaks at *m*/*z* 151.0214, 273.1736, and 345.2300 are related with 3-ADON degradation products by SMCD 2220-01 or its fragments or metabolite of SMCD 2220-01 induced by 3-ADON (Fig. 6d). A peak at *m*/*z* 345.2300 might be a fragment ion of the deoxynivalenol-3-sulfate, which may be result of deacetylation of 3-ADON (converted to DON) and then sulfation of DON by SMCD 2220-01.

**Fig. 7:**
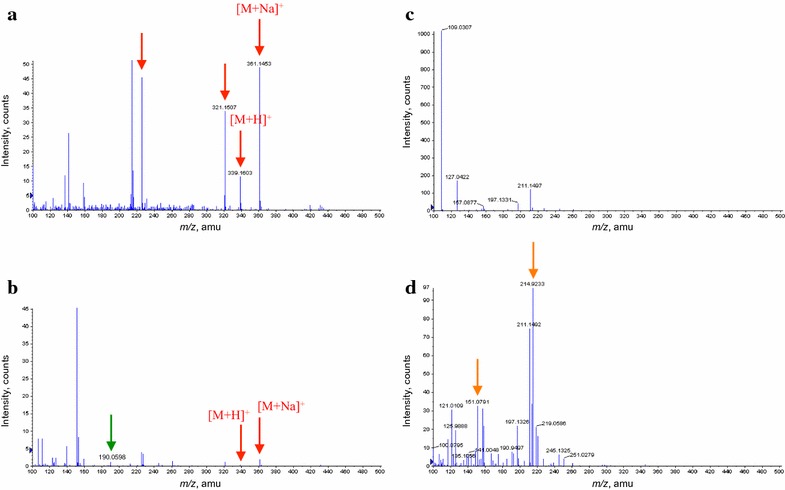
Representative mass spectra of extracts culture filtrates analyzed by ESI–TOF–MS in positive-ion mode for 15-ADON treatments and controls. 15-ADON in PDB (**a**), SMCD 2220-01 treated with 15-ADON in PDB (**b**), PDB (**c**), and SMCD 2220-01 in PDB (**d**)

The extracts culture filtrates of both 15-ADON in PDB and SMCD 2220-01 treated with 15-ADON in PDB showed peaks of [M+H]^+^ at *m*/*z* 339.1603 and [M+Na]^+^ at *m*/*z* 361.1453 in positive-ion mode (Fig. 7a, b), identified as 15-ADON. It is likely that peaks at *m*/*z* 225.2032 are related with 15-ADON, which might be fragments of 15-ADON. The very weak peak at *m*/*z* 190.0598 seems to relate with 15-ADON degradation product by SMCD 2220-01. The peaks at *m*/*z* 151.0791 and 214.9233 seem to relate with metabolite(s) of SMCD 2220-01 compared with PDB (Fig. 7c, d).

## Discussion

The presence of mycotoxins is inherent to many grains, food and feed products worldwide (Vanhoutte et al. 2016). For many years the research community focused on the occurrence of singular mycotoxins but nowadays scientific interest shifts to studies involving multiple mycotoxins, in particularly for *Fusarium* species and associated mycotoxins in cereal grain, food and feed (Kim and Vujanovic 2016). Microbial detoxification or biotransformation of mycotoxins includes different types of reaction, such as acetylation, glucosylation, ring cleavage, hydrolysis, deamination, and decarboxylation (McCormick 2013).

Biotransformation or biodegradation of ZEN has been reported by *Bacillus* spp. (Xu et al. 2016) and *Pseudomonas* spp. strains (Tan et al. 2014). However, metabolic products of ZEN were not identified from those strains. A possible pathway might comprise of cleavage of a ring structure followed by decarboxylation for complete degradation of ZEN as shown by *Bacillus* strains (Tinyiro et al. 2011). Besides bacteria, several fungi were known as degrader of ZEN by producing different metabolites of ZEN: *Rhizopus* spp. producing *α-*zearalenol and *β*-zearalenol (Brodehl et al. 2014); *Aspergillus ochraceous* and *Aspergillus niger* producing *α*-zearalanol and *β*-zearalanol (El-Sharkawy and Abul-Hajj 1988b); *Cunninghamella bainieri* producing 2,4-dimethoxyzearalenone and 2-methoxyzearalenone (El-Sharkawy and Abul-Hajj 1988b); *Rhizopus arrhizus* producing zearalenone 4-sulfate (El-Sharkaway et al. 1991); *Thamidium elegans* and *Mucor bainieri* producing zearalenone-4-*β*-d-glucoside (El-Sharkawy and Abul-Hajj 1987). However, some of ZEN metabolic products such as *α-*zearalenol, *α*-zearalanol, and *β*-zearalanol showed higher toxicity and higher relative estrogenicity than ZEN (Shier et al. 2001).

Divergently, a fungal mycoparasite *Clonostachys rosea* showed the ability to detoxify ZEN to a ring cleavage product, 1-(3,5-dihydroxy-phenyl)-10′-hydroxy-1′*E*-undecene-6′-one (Kakeya et al. 2002; Takahashi-Ando et al. 2002). Furthermore, it was shown that the detoxification of ZEN by zearalenone hydrolase of *C. rosea* is crucial for the successful mycoparasitism against *F. graminearum* (Kosawang et al. 2014).

**Fig. 8:**
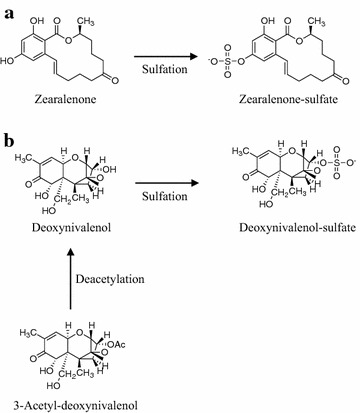
Proposed (partial) pathway of ZEN degradation (**a**) and DON and 3-ADON degradation (**b**) by *S. mycoparasitica* SMCD 2220-01

In this study, *S. mycoparasitica* SMCD 2220-01 was efficient in decreasing the level of multiple *Fusarium* mycotoxins including ZEN by 97%, DON by 89%, 15-ADON by 72%, and 3-ADON by 58% revealed by TLC and HPLC–ESI–HRMS. The transformant of ZEN by SMCD 2220-01 could be identified as zearalenone sulfate as revealed by LC–ESI–HRMS analysis (Fig. 8a). Furthermore, deoxynivalenol sulfate as transformed DON and 3-ADON metabolic product by SMCD 2220-01 was also detected (Fig. 8b). These findings demonstrate that the mycoparasite not only parasites on the host, but also degrades *Fusarium* mycotoxins or transforms them to less toxic compounds. Indeed, it was reported that modification or transformation of the aromatic ring of ZEN resulted in a remarkably decreased estrogenic activity compared to non-transformed ZEN (Drzymala et al. 2015). Therefore, biodegradation of the mycotoxins by SMCD 2220-01 could be considered as a process of detoxification for ZEN and DON, minimazing *Fusarium* mycotoxins in field crops and preventing reduction in grade and end-use quality of grains, food and feed.

Zearalenone sulfate, as a ZEN degradation product by SMCD 2220-01, is different from the ZEN degradation product by *C. rosea*. The differences in a type of degradation products may be due to distinct extracellular enzymes employed. In order to illuminate the metabolic mechanisms of degradation and/or detoxification of ZEN and DON by SMCD 2220-01, more research should be done on the specific fungal enzymes and their abilities to biotransform *Fusarium* mycotoxins to less toxic or non-toxic metabolites (Kluger et al. 2015; Ji et al 2016). The expression of genes coding for degrading enzymes involved in reduction of mycotoxins, are merited. It is important to note a comparative advantage of SMCD 2220-01 properties, as specific-mycoparasite to mycotoxigenic Fusaria, as source of the enzymes and vital biotechnological tool to mycotoxin biodegradation and biodetexofication (Kim and Vujanovic 2016). The *Sphaerodes* mycoparasitic properties might be tightly connected to its biotrophic mycoparasite lifestyle and resistance to *Fusarium*, which implies that SMCD 2220-01 could be used as an efficient biocontrol agent. A full understanding and appropriate application of this mycoparasite should be helpful towards the development of novel microbiological solutions for reducing mycotoxin contamination-related to *Fusarium* infection in grains and for increasing mycotoxin detoxification in foods and feeds.

## Abbreviations

AUR: aurofusarin
3-ADON: 3-Acetyldeoxynivalenol
15-ADON: 15-Acetyldeoxynivalenol
DON: deoxynivalenol
HPLC–ESI–HRMS: high performance liquid chromatography–electrospray ionization–high resolution mass spectrometry
PDB: potato dextrose broth
TLC: thin layer chromatography
ZEN: zearalenone
